# Micro-Photographs in Histology, Normal and Pathological

**Published:** 1876-12

**Authors:** 


Micro-Photographs in Histology, Normal and Pathological. By Carl Siler, M.D., in conjunction with J. Gibbons Hunt, M.D., and Joseph G. Richardson, M.D. Philadelphia: J. H. Coates & Co., Publishers. 1876.

This, a monthly publication of micro-photographs of physiological and pathological histology, bids fair to serve as a valuable assistant in the prosecution of studies in pathological anatomy. We have upon our table the sixth number of volume one, and regret that the file is not complete. In the number before us we find photographs of the microscopical appearance of amyloid infiltration of the kidney; hyaline casts in advanced stage of Bright's disease; uric acid and triple phosphates. Following them is a full and definite explanation of the plates, with an epitome of the morbid conditions in which each product is found, and the manner of detecting them by microscopical means. It is a neat pamphlet, and the gentlemen getting it up deserve credit and commendation.

